# Proximate grassland and shrub-encroached sites show dramatic restructuring of soil bacterial communities

**DOI:** 10.7717/peerj.7304

**Published:** 2019-07-19

**Authors:** Xingjia Xiang, Sean M. Gibbons, He Li, Haihua Shen, Haiyan Chu

**Affiliations:** 1 Anhui Province Key Laboratory of Wetland Ecological Protection and Restoration, School of Resources and Environmental Engineering, Anhui University, Hefei, China; 2 Institute for Systems Biology, Seattle, WA, USA; 3 School of Geography, Geomatics and Planning, JiangSu Normal University, Xuzhou, China; 4 State Key Laboratory of Vegetation and Environmental Change, Institute of Botany, Chinese Academy of Sciences, Beijing, China; 5 State Key Laboratory of Soil and Sustainable Agriculture, Institute of Soil Science, Chinese Academy of Sciences, Nanjing, China

**Keywords:** Shrub encroachment, Bacterial community, Grassland ecosystem, Niche filtering, Sequencing

## Abstract

**Background:**

Changes in aboveground community composition and diversity following shrub encroachment have been studied extensively. Recently, shrub encroachment was associated with differences in belowground bacterial communities relative to non-encroached grassland sites hundreds of meters away. This spatial distance between grassland and shrub sites left open the question of how soil bacterial communities associated with different vegetation types might differ within the same plot location.

**Methods:**

We examined soil bacterial communities between shrub-encroached and adjacent (one m apart) grassland soils in Chinese Inner Mongolian, using high-throughput sequencing method (Illumina, San Diego, CA, USA).

**Results:**

Shrub-encroached sites were associated with dramatic restructuring of soil bacterial community composition and predicted metabolic function, with significant increase in bacterial alpha-diversity. Moreover, bacterial phylogenic structures showed clustering in both shrub-encroached and grassland soils, suggesting that each vegetation type was associated with a unique and defined bacterial community by niche filtering. Finally, soil organic carbon (SOC) was the primary driver varied with shifts in soil bacterial community composition. The encroachment was associated with elevated SOC, suggesting that shrub-mediated shifts in SOC might be responsible for changes in belowground bacterial community.

**Discussion:**

This study demonstrated that shrub-encroached soils were associated with dramatic restructuring of bacterial communities, suggesting that belowground bacterial communities appear to be sensitive indicators of vegetation type. Our study indicates that the increased shrub-encroached intensity in Inner Mongolia will likely trigger large-scale disruptions in both aboveground plant and belowground bacterial communities across the region.

## Introduction

Increased cover, abundance and dominance of shrub species in grasslands have been widely reported, with 10–20% of arid and semiarid grassland area undergoing shrub encroachment across the world (Van Vegten, 1984; Jackson et al., 2002; Maestre et al., 2009). Multiple factors appear to trigger shrub encroachment, including grazing pressure (Coetzee et al., 2008), climate change (i.e., global warming, elevated CO_2_, nitrogen deposition; Archer, Schimel & Holland, 1995) and wildfire frequency (Scholes & Archer, 1997). Around 330 million ha of grassland were subject to shrub invasion in xeric western states of United States (Knapp et al., 2008). A total of 13 million ha of savanna are undergoing shrub encroachment in South Africa (Eldridge et al., 2011). Moreover, similar conditions were demonstrated in many other areas of the world (e.g., Eurasian and Australian grasslands; Zhang et al., 2006; Rivest et al., 2011; Chen et al., 2015). Shrub encroachment significantly affects the livestock industry, which also has important ecological repercussions in arid and semiarid grasslands.

Shrub encroachment into native grassland results in a loss of biodiversity that can affect ecosystem functioning (Throop & Archer, 2008). Areas undergoing encroachment are characterized by patchy vegetation, with clusters of shrubs and areas dominated by grasses. Shrub and grass patches differ in above-ground community composition, overall primary productivity, plant allocation, and rooting depth (Trumbore, 1997; Briggs et al., 2005; McClaran et al., 2008; Meyer, Wiegand & Ward, 2009), leading to the long-term profound effects of encroachment on grassland ecosystems, including changes in soil erosion, soil moisture (SM), soil carbon, soil pH, energy cycling, soil aeration, soil nitrogen contents, and soil faunal communities (Lett & Knapp, 2003; Smith & Johnson, 2003; Breshears, 2006; Knapp et al., 2008; McKinley & Blair, 2008). The impacts induced by encroachment are not always coincident, sometimes leading to a decrease (Gómez-Rey et al., 2013) or an increase (Soliveres & Eldridge, 2014) in aboveground plant productivity. Shrub encroachment is often related to soil nutrient accumulation (“islands of fertility”; Reynolds et al., 1999; Peng et al., 2013) due to litterfall and nitrogen fixation (Schlesinger et al., 1990; Hibbard et al., 2001).

It is plausible that complicated feedback mechanisms present among aboveground vegetation, belowground properties, and microbial communities (Hart et al., 2005). Soil microorganisms play crucial roles in belowground ecosystems, serving as catalysts for nutrient transformations, forming mutualistic relationship with plants to improve host health, and working as engineers to maintain soil structure (Hart et al., 2005; Paul & Clark, 1996). Shrub encroachment triggers large shifts in plant and soil properties, which may directly and indirectly affect soil microbial communities. Soil properties, such as soil carbon content (Zhang et al., 2014) and pH (Griffiths et al., 2011) significantly affect microbial community structure. Plant litterfall and root exudates provide nutrients to feed soil heterotrophic microbes (Staddon et al., 2003). Previous studies have found that revegetation significantly affected soil microbial biomass and community structure (Yannarell, Menning & Beck, 2014; Bragazza et al., 2015). Shrub encroachment significantly altered soil microbial communities, soil respiration, extracellular enzyme activity, and denitrification potential in subtropical marshes (Ho & Chambers, 2019). Previously, we found dramatic shifts in soil bacterial communities associated with shrub encroachment relative to distant grassland soils (i.e., >500 m), without exploring associations between vegetation type and soil properties within the same sampling location (Xiang et al., 2018).

In China, shrubs have occupied more than 5.1 million ha grassland in Inner Mongolian of China (Chen et al., 2015). A better understanding of bacterial community structure in shrub-encroached soils is crucial for clarifying the influence of encroachment on grassland ecosystem functioning. In this study, we focus on soil bacterial community composition within shrub-dominated and adjacent grassland-dominated patches (one m apart) in the same sampling site. In particular, we addressed two main questions: (i) how encroachment affects soil bacterial community composition and diversity; and (ii) what are the main factors driving soil bacterial communities following shrub encroachment.

## Materials and Methods

### Site description and sample collection

The study area was selected in a high-density shrub-encroached grassland (42°57′N, 112°43′E; 1,208 m; Fig. S1), located in Inner Mongolia, China. The average annual temperature is 5.1 °C and the mean precipitation is 195 mm in this region (Chen et al., 2015). The dominated grass is *Cleistogenes songorica* across the region, but *Caragana microphylla* is encroaching (Chen et al., 2015). Soil samples were collected on the 10th of August, 2016. We identified ten shrub-encroached sample plots to include in this study. The selected sites were more than 500 m away from each other. At each site (10 × 10 m), the encroachment soils were sampled under five shrub patches (the nearest to the four vertices and the center of a plot) with 0–10 cm depth and mixed as one sample. The control non-encroached soils were collected one m away from the five shrub canopies with 0–10 cm depth and mixed as one sample (Fig. S1). In total, 10 from control grassland soils and 10 from adjacent encroached soils were collected for further study. The soils were fully mixed and sieved, and then transported refrigerated to the lab within 24 h. The soils were divided into two parts: one part was stored at 4 °C for biogeochemical analysis and the other was stored at −20 °C for DNA extraction.

### Sample pretreatment

Measurement of soil properties, DNA extraction, and amplicon library preparation are described in the Supplemental Information.

### Processing of sequence data

The raw data were processed by QIIME (v.1.9.0; Caporaso et al., 2010). The sequences were clustered into operational taxonomic units (OTUs; 97% identity) with UCLUST (Edgar, 2010). Chimeric and singleton OTUs were removed prior to downstream analysis. The default setting was used to select the representative sequence (i.e., most abundant sequence) for each OTU, which was assigned taxonomic annotations using the UCLUST (Edgar, 2010) and aligned by PyNAST (Caporaso et al., 2010). To normalize for sampling depth, random subsets of 26,000 reads per sample (the lowest sequence read depth across the study) were used to calculate bacterial alpha- and beta-diversities.

### Statistical analysis

Phylogenetic diversity (PD) was estimated by Faith’s index (Faith, 1992). Pairwise *t*-test was performed to show differences in relative abundance of dominant bacterial phyla and alpha-diversity. Pearson correlation was used to test relationships between bacterial alpha-diversity and soil properties. Linear discriminant analysis effect size (LEfSe) was used to identify bacterial taxa that differed significantly between treatments (default setting; Segata et al., 2011). Non-metric multidimensional scaling and Analysis of Similarity (ANOSIM; permutations = 999) were performed to distinguish the differences in bacterial community composition between treatments by using the vegan package (v.2.0-2) in R software. The correlation between variables (i.e., soil properties and spatial distance) and soil bacterial community composition were analyzed by Mantel tests (permutations = 999). Multicollinearity of soil properties was tested by the variance inflation factor (VIF; Zuur, Leno & Elphick, 2010), and those properties with the VIF values < 3 were selected for canonical correspondence analysis (CCA).

The nearest taxon index (NTI) and beta nearest taxon index (betaNTI) were performed using the picante package (Purcell et al., 2007) and Phylocom 4.2 (Hardy, 2008), respectively, to analyze soil bacterial phylogenetic structure. The NTI measures the mean nearest taxon distance among individuals to estimate the phylogenetic dispersion of the community (Webb, 2000). More positive or negative NTI values indicate phylogenetic clustering or overdispersion, respectively (Webb, 2000). BetaNTI values between −2 and 2 suggested stochastic process (neutral assembly) while the values above 2 or below −2 indicated deterministic processes (niche assembly, Stegen et al., 2012). Co-occurrence networks were generated in R using the “WGCNA” package (Langfelder & Horvath, 2012). We adjusted all *P*-values (cutoff as 0.001) by using the Benjamini and Hochberg false discovery rate for multiple testing (Benjamini, Krieger & Yekutieli, 2006). The network nodes defined as network hubs (*z*-score > 2.5; *c*-score > 0.6), module hubs (*z*-score > 2.5; *c*-score < 0.6), connectors (*z*-score < 2.5; *c*-score > 0.6), and peripherals (*z*-score < 2.5; *c*-score < 0.6) referring to their roles in network structure (Poudel et al., 2016). Network hubs are those OTUs that are highly connected both in general and within a module. Module hubs and connectors are OTUs that are highly connected only within a module and only link modules, respectively. Peripherals are defined as OTUs that have few links to other species. The bacterial metabolic function was predicted by phylogenetic investigation of communities by reconstruction of unobserved states (PICRUSt) according to KEGG database (Langille et al., 2013).

## Results

### Soil chemistry

Compared to non-encroached grassland soils, shrub-encroached soils were associated with higher content of NO_3_^−^, total nitrogen (TN), total carbon (TC), dissolved organic carbon (DOC), soil organic carbon (SOC), and total phosphorus (Table S1). However, shrub encroachment showed little effect on other soil properties, such as soil pH, NH_4_^+^ content and SM relative to control in this study (Table S1).

### Bacterial alpha-diversity

**Figure 1:**
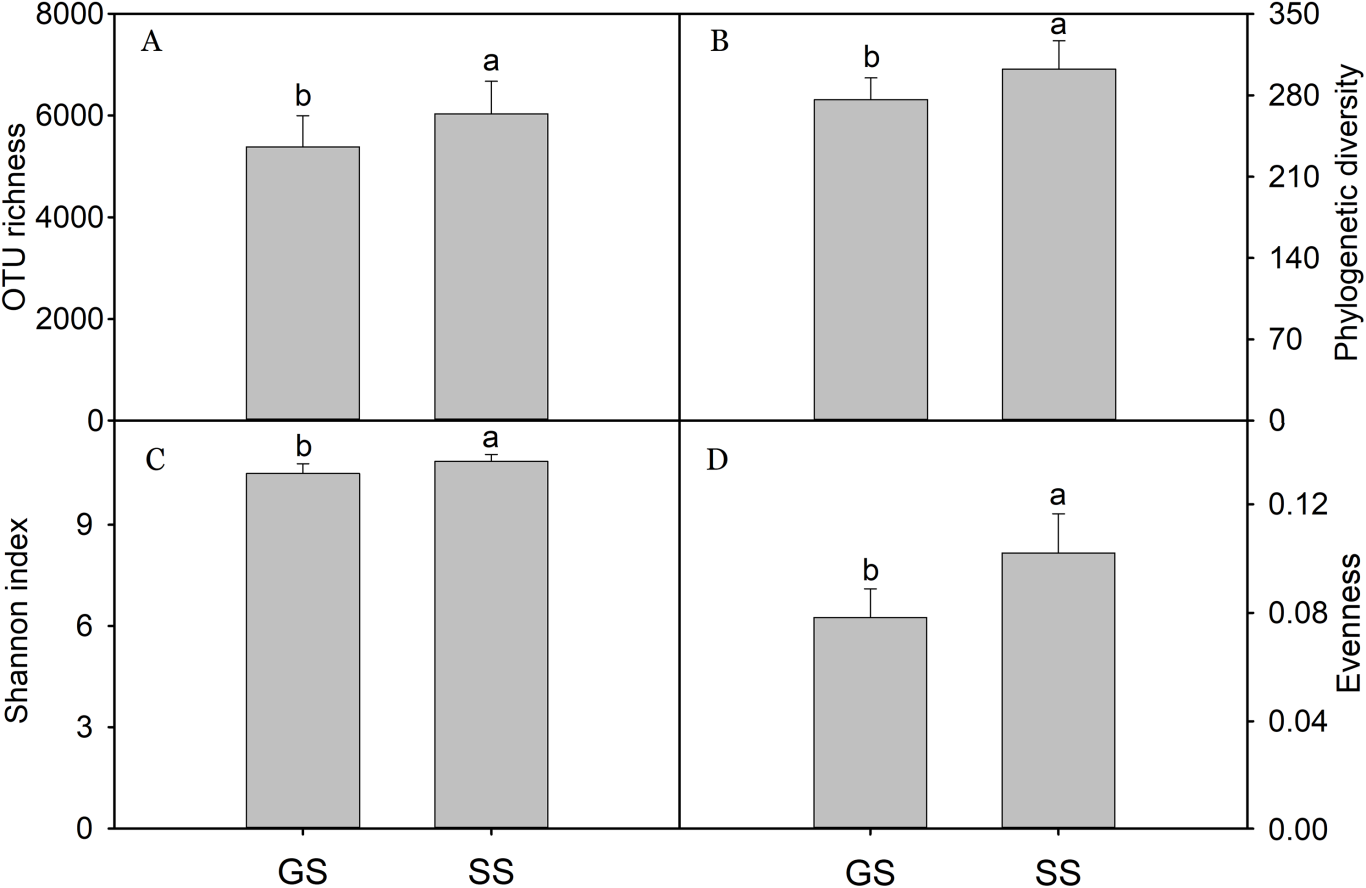
Soil bacterial diversity. Bacterial alpha-diversity calculated at a rarefaction depth of 26,000 randomly selected sequences per sample. (A) bacterial OTU richness; (B) bacterial phylogenetic diversity; (C) bacterial Shannon index; (D) bacterial evenness. Bars represent mean; error bars denote standard deviation; letters above bars represents significant differences from pairwise *t*-test (*P* < 0.05). GS: control grassland soil; SS: shrub-encroached soil.

A total of 966,631 quality bacterial sequences was obtained with 26,037–68,261 (mean 48,332) sequences per sample. In this study, bacterial alpha-diversity included OTU richness, Shannon index, evenness, and PD, which was calculated by randomly selected subsets of 26,000 reads per sample. Generally, encroached sites had significant higher alpha-diversity relative to grassland sites (Fig. 1). Bacterial OTU richness was positively correlated with NO_3_^−^, DOC, TC, TP, and SOC; PD was positively correlated with NO_3_^−^, DOC, TC, and SOC; the Shannon index was positively correlated with NO_3_^−^, TC, TP, and SOC; evenness was positively correlated with NO_3_^−^, TN, TC, and SOC (Table 1).

**Table 1:** The correlation between bacterial alpha-diversity and soil properties.

Variables	OTU	PD	Shannon	Evenness
Soil pH	0.276	0.327	0.077	−0.164
Soil moisture (%)	0.167	0.109	0.315	−0.045
NH_4_^+^-N (mg/kg)	−0.300	−0.146	0.276	0.413
NO_3_^−^-N (mg/kg)	0.502^*^	0.532^*^	0.475^*^	0.563^*^
Dissolved organic C (mg/kg)	0.471^*^	0.483^*^	0.402	0.400
Dissolved organic N (mg/kg)	0.131	0.148	0.176	0.138
Total carbon (mg/g)	0.486^*^	0.495^*^	0.491^*^	0.485^*^
Total nitrogen (mg/g)	0.389	0.394	0.362	0.474^*^
Total phosphorus (mg/g)	0.462^*^	0.421	0.493^*^	0.255
Soil inorganic carbon (mg/g)	−0.058	0.019	−0.014	0.248
Soil organic carbon (mg/g)	0.582^*^	0.586^*^	0.574^*^	0.601^*^

**Notes:**

Significant correlations are shown in bold (*P* < 0.05).

* *P* < 0.05; OTU, operational taxonomic unit; PD, phylogenetic diversity.

### Bacterial community structure

**Figure 2:**
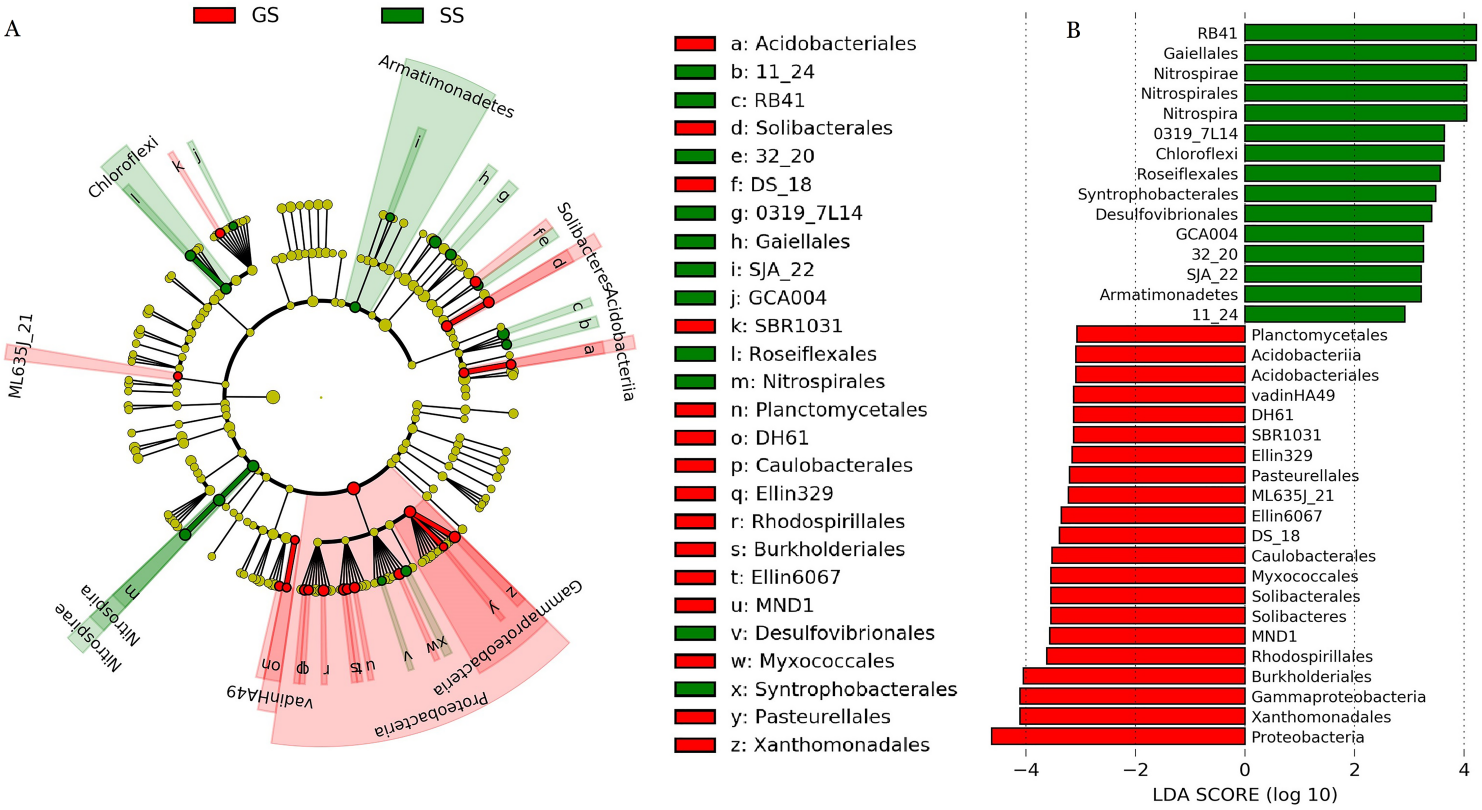
LEfSe analysis of soil bacterial biomarkers associated with vegetation type. (A) cladogram representing the taxonomic hierarchical structure of the phylotype biomarkers identified between two vegetation types. Each filled circle represents one biomarker. Red, phylotypes statistically overrepresented in grassland soil; green, phylotypes overrepresented in shrub-encroached soil; yellow, phylotypes for which relative abundance is not significantly different between the two vegetation types. (B) Identified phylotype biomarkers ranked by effect size and the alpha value was <0.05.

The dominant soil bacterial phyla (i.e., relative abundance > 1%) across all samples were Actinobacteria (27.3%), Acidobacteria (23.1%), Proteobacteria (23.0%), Chloroflexi (6.0%), Planctomycetes (4.7%), Gemmatimonadetes (2.8%), Firmicutes (2.7%), Bacteroidetes (2.6%), and Nitrospirae (2.4%) (Fig. S2). Compared to control grassland soils, the relative abundance of Proteobacteria showed significantly lower in encroached sites (Fig. S3). Compared to control, encroachment was associated with higher relative abundance of Chloroflexi and Nitrospirae (Fig. S3). LEfSe analysis showed that bacteria in one phylum (i.e., Proteobacteria), five classes (i.e., *Acidobacteriia*, *ML635J_21*, *vadinHA49*, *Solibacteres*, and *Gammaproteobacteria*) and 15 orders (i.e., *Acidobacteriales*, *Solibacterales*, *Planctomycetales*, *Caulobacterales*, *Rhodospirillales*, *Burkholderiales*, etc) were significantly more abundant in control soils. Bacteria from two phyla (i.e., Nitrospirae and Armatimonadetes), two classes (i.e., *Nitrospira* and *Chloroflexi*) and 11 orders (i.e., *Gaiellales*, *Roseiflexales*, *Nitrospirales*, *Syntrophobacterales*, *Desulfovibrionales*, etc) were significantly more abundant in shrub-encroached soils (Fig. 2).

**Figure 3:**
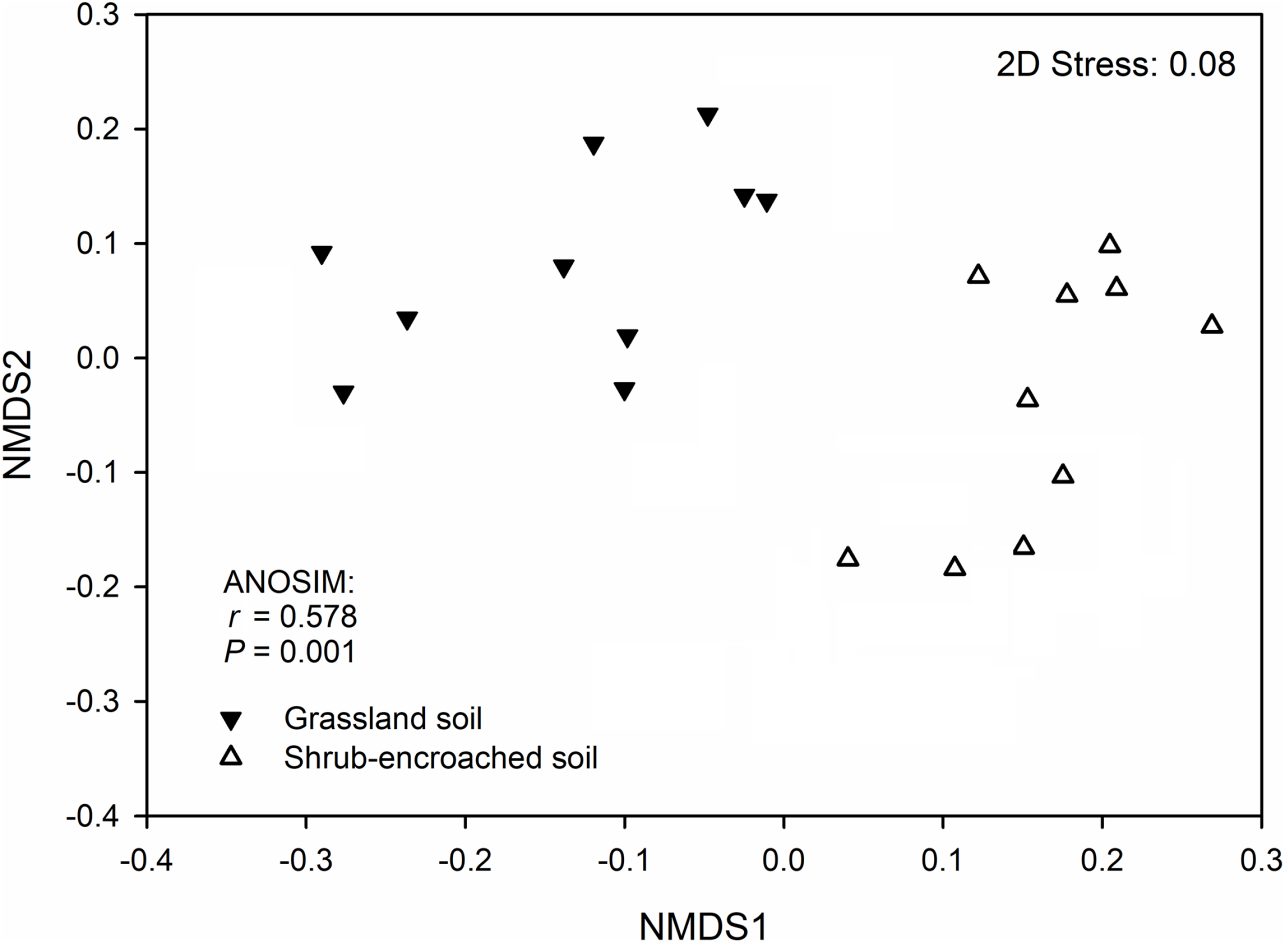
Non-metric multidimensional scaling (NMDS) plot. Non-metric multidimensional scaling (NMDS) plot showing bacterial community composition in control grassland and shrub-encroached soils of Inner Mongolia.

**Figure 4:**
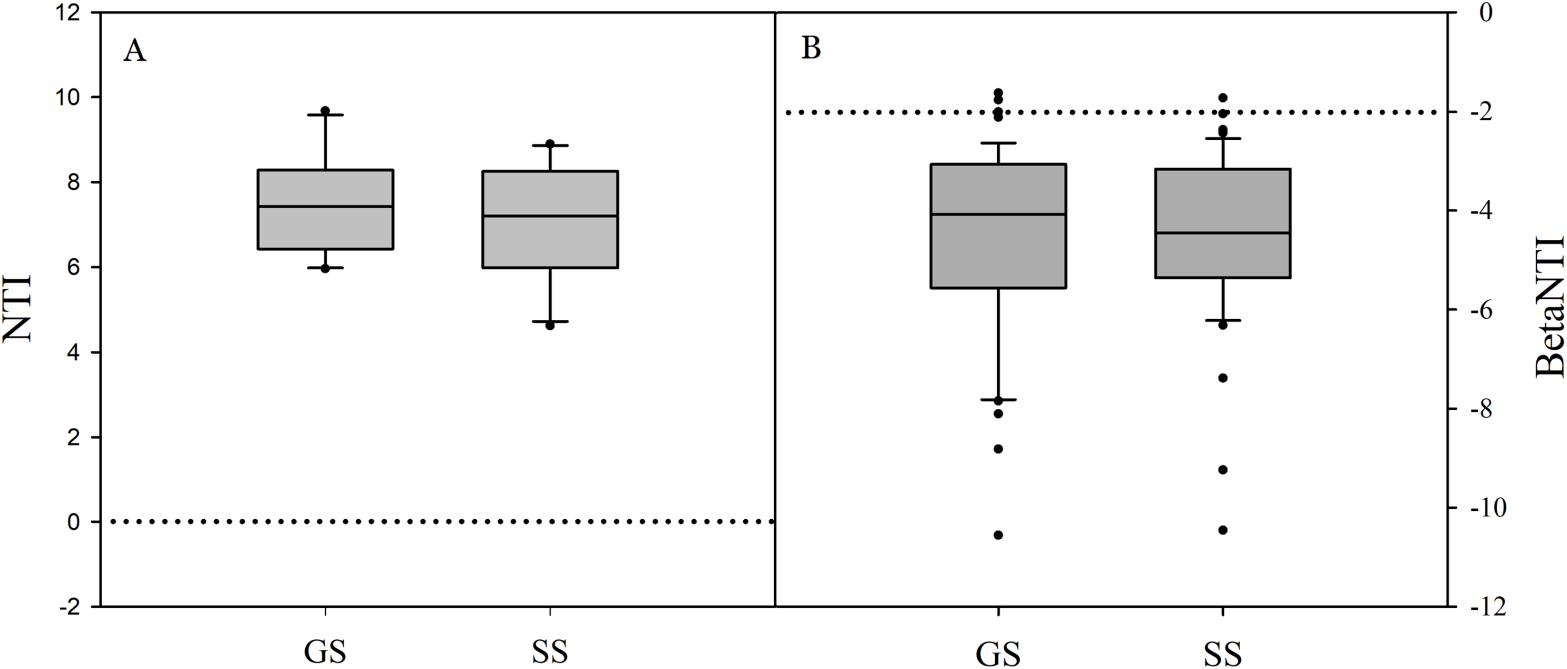
The values of nearest taxon index (NTI; A) and beta nearest taxon index (betaNTI; B) in grassland and shrub-encroached soils. GS: control grassland soil; SS: shrub-encroached soil.

Significant differences in soil bacterial community compositions were found between shrub-encroached and grassland sites (ANOSIM: *P* = 0.001; Fig. 3). The NTI values showed positive (i.e., >0; *P* = 0.001) for all samples, indicating that bacterial phylogenetic structure showed clustering in both encroached and control soils (Fig. 4). Almost all betaNTI scores for bacterial communities were below −2, which suggested that deterministic assembly dominated soil bacterial community dynamics in both grassland and shrub-encroached soils (Fig. 4). A correlation network was built at bacterial genus level. There was a larger proportion of positive than negative correlations between genera in soils (Fig. S4A). Compared to grassland soils, shrub-encroached soil showed higher proportion of correlation network hubs (Fig. S4B), suggesting that bacterial community in shrub-encroached soils might be more interconnected than grassland soils.

Mantel tests demonstrated that soil bacterial community composition showed significant correlation with soil pH, SM, NO_3_^−^, DOC, TC, TP, and SOC (Table 2; *P* < 0.05 in all cases). Among these variables, SOC content (*P* = 0.002) had the strongest correlation with soil bacterial community composition. However, spatial distance showed little correlation with bacterial community composition (*P* = 0.181; Table 2). CCA further demonstrated that SOC was the primary driver affecting soil bacterial community composition (Fig. S5).

**Table 2:** Mantel test showing the effect of soil properties on bacterial community composition.

Variables	Mantel test	
	*r*	*P*
Soil pH	**0.295**	**0.003**
Soil moisture (%)	**0.218**	**0.012**
NH_4_^+^-N (mg/kg)	0.198	0.053
NO_3_^−^-N (mg/kg)	**0.282**	**0.017**
Dissolved organic C (mg/kg)	**0.295**	**0.006**
Dissolved organic N (mg/kg)	0.090	0.183
Total carbon (mg/g)	**0.196**	**0.049**
Total nitrogen (mg/g)	0.114	0.164
Total phosphorus (mg/g)	**0.185**	**0.041**
Soil inorganic carbon (mg/g)	0.168	0.079
Soil organic carbon (mg/g)	**0.412**	**0.002**
Spatial distance (m)	0.068	0.181

**Note:**

Comparing differences between samples in bacterial community composition to differences between samples in variables (i.e., soil properties and spatial distance) by Mantel tests. Significant correlations are shown in bold (*P* < 0.05).

### The predicted metabolic function

**Figure 5:**
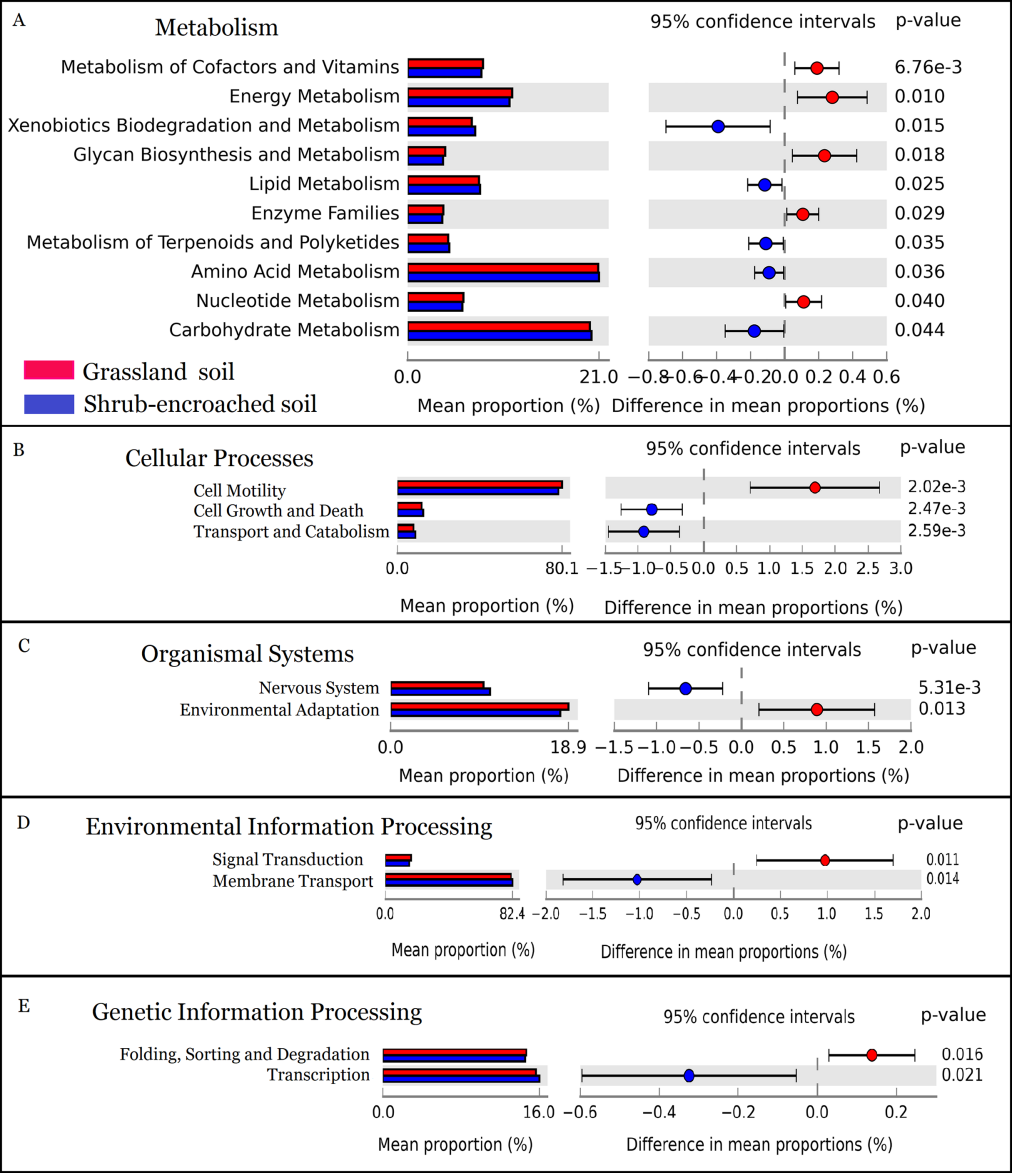
The predicted metabolic function profiles of bacterial community. Variation of metabolic function profiles of bacterial community in grassland and shrub-encroached soils analyzed by PICRUSt. (A) Metabolism; (B) Cellular Processes; (C) Organismal Systems; (D) Environmental Information Processing; (E) Genetic Information Processing.

The metabolic function of bacterial community was predicted by PICRUSt. A total of 328 predicted functional genes were detected in this study. More than 89% of total sequences belonged to categories of metabolism (52.2%), genetic information processing (15.8%), environmental information processing (13.3%), and organismal systems (8.35%) in soils, according to the KEGG database. Compared to controls, shrub encroachment was associated with significant differences in potential functions of the soil bacterial community (Fig. S6). Metabolism of cofactors and vitamins, energy metabolism, glycan biosynthesis and metabolism, enzyme families, and nucleotide metabolism were enriched in grassland soil, while xenobiotics biodegradation and metabolism, lipid metabolism, metabolism of terpenoids and polyketides, amino acid metabolism, and carbohydrate metabolism were enriched in shrub-encroached soils (Fig. 5). The relative abundances of sequences associated with cell motility, environmental adaptation, signal transduction, and protein folding, sorting and degradation were enriched in grassland soils (Fig. 5). The sequences related to cell growth and death, transport and catabolism, nervous system, membrane transport, and transcription were enriched in shrub-encroached soils (Fig. 5).

## Discussion

In this study, encroachment triggered significant changes in soil bacterial community composition (Fig. 3), and an apparent increase in bacterial alpha-diversity (Fig. 1), which is consistent with other studies showing that aboveground vegetation triggers a profound influence on belowground bacterial communities (Bragazza et al., 2015; Gellie et al., 2017). Recently, we found dramatic shifts in soil bacterial communities associated with shrub encroachment relative to distant grassland soils (i.e., >500 m; Xiang et al., 2018), which is consistent with the current study, which shows a restructuring of bacterial communities between shrub-encroached and adjacent (one m apart) grassland soils, indicating that soil bacterial community appears to be sensitive indicator of plant cover type. In addition, bacterial alpha-diversity showed significant correlations with soil nutrient levels (e.g., SOC, etc; Table 1), which increased following shrub encroachment (Table S1; Bragazza et al., 2015), indicating that elevated soil nutrients might reduce competition within bacterial communities and allow rare species to persist, leading to an increase in soil bacterial alpha-diversity (Xiang et al., 2018). Our results go beyond these findings by showing that the predicted metabolic function differed significantly between grassland and shrub-encroached soils (Fig. 5; Fig. S6), suggesting that shrub encroachment likely triggers significant shifts in grassland ecosystem functioning.

Similarly, we found strong evidence for reproducible environmental filtering in encroached and control soils in this study (Fig. 4), indicating that different vegetation types were associated with specific belowground bacterial communities (Wallenstein, McMahon & Schimel, 2007; Chu et al., 2016). Environmental filtering may include access to specific carbon sources and changes in soil chemistry (Prescott & Grayston, 2013). Previous research also showed substantial differences in bacterial community compositions among four vegetation types (Gibbons et al., 2017), providing evidence for dynamic and complex feedbacks between aboveground plant and belowground bacterial community structure (Shi et al., 2015; Gibbons et al., 2017).

Soil pH has been demonstrated to be a dominant factor in driving belowground bacterial community composition (Baker et al., 2009). However, compared to adjacent grassland soils (one m apart), shrub encroachment was not predominantly related to the shift in soil pH. The primary influence of pH on bacterial community composition was not detectable in this study, possibly induced by limited variation of pH range between grassland and nearby shrub-encroached soils. In this study, shrub-encroached soil was strongly related to an increase in SOC content, which was the primary factor in explaining the variance in bacterial community composition across sites (Table 2; Fig. S5). Sul et al. (2013) also demonstrated that SOC was the most important factor to explain the differences in the bacterial community composition in a tropical agricultural ecosystem. A prior study showed that plant communities altered SOC concentrations to indirectly affect belowground bacterial community composition (Liu et al., 2014). In addition, soil carbon fraction might be a crucial factor in shaping microbial communities (Zhou et al., 2012). Plants may influence bacterial communities by determining the quantity and quality of the litterfall supply (Wallenstein, McMahon & Schimel, 2007) and/or by releasing photosynthetic products into the soil (Staddon et al., 2003). Shrubs may contribute qualitatively different carbon inputs (i.e., litterfall, root exudate, etc; Schlesinger et al., 1990; Archer, Schimel & Holland, 1995) to soils and thereby influence soil bacterial communities. Therefore, we speculate that shrub-mediated changes in SOC appear to be primary responsible for changes in composition of bacterial community.

A prior study demonstrated that shrub expansion was associated with enhanced N availability, which in turn facilitated shrub expansion and increased shrub patch density (Chu & Gorgan, 2010). We found that soil NO_3_^−^ content showed significant enrichment in shrub-encroached sites (Table S1). Moreover, shrub encroachment was related to elevated relative abundance of *Nitrospira*, which performs soil nitrification process (Daims et al., 2015) (Fig. 2), indicating that the higher relative abundance of *Nitrospira* might lead to the accumulation of soil NO_3_^−^ following shrub encroachment (Xiang et al., 2018). Soil NH_4_^+^ concentrations did not differ between grassland and shrub-encroached sites. Thus, enhanced N availability in shrub encroached sites appears to be induced by elevated soil NO_3_^−^, which may act as a positive feedback on shrub encroachment (Chu & Gorgan, 2010).

Overall, we propose a possible feedback among vegetation, soil properties, and bacterial community following encroachment based on our results, whereby: (1) shrub encroachment increases soil organic matter (e.g., litterfall, etc; Schlesinger & Pilmanis, 1998; Kurc & Small, 2004), which (2) activates soil microbes and alters soil nutrient cycling, and (3) greater resulting N availability facilitates shrub expansion and increased shrub densities around established shrub patches (Chu & Gorgan, 2010).

## Conclusions

This study demonstrated that shrub-encroached soils were associated with significant increase in bacterial alpha-diversity and dramatic restructuring of bacterial community composition. Environmental filtering (e.g., SOC content, etc) appears to mediate the influence of vegetation type on belowground microbial communities. The results of predicted metabolic function suggested that shrub encroachment might trigger large-scale disruptions of grassland ecosystem functioning. This work helps to further refine our knowledge of how shrub encroachment affects bacterial community structure in grassland ecosystems. However, we did not investigate the effect of encroachment on soil fungal communities, which might be more important for carbon cycling and closely related to changes in vegetation. This limitation should be addressed in future studies.

## Supplemental Information

10.7717/peerj.7304/supp-1 Supplemental Information 1 Raw data for soil properties in grassland and shrub-encroached soils.

10.7717/peerj.7304/supp-2 Supplemental Information 2 Supplemental Materials and Methods, Table, and Figures.

## Additional Information and Declarations

### Data Availability

The following information was supplied regarding data availability: The raw data is available at the Sequence Read Archive (SRA) of NCBI under the accession number SRP136091.

## References

[ref-1] Archer S, Schimel DS, Holland EA (1995). Mechanisms of shrubland expansion: land use, climate or CO2?. Climatic Change.

[ref-2] Baker KL, Langenheder S, Nicol GW, Ricketts D, Killham K, Campbell CD, Prosser JI (2009). Environmental and spatial characterisation of bacterial community composition in soil to inform sampling strategies. Soil Biology and Biochemistry.

[ref-3] Benjamini Y, Krieger AM, Yekutieli D (2006). Adaptive linear step-up procedures that control the false discovery rate. Biometrika.

[ref-4] Bragazza L, Bardgett RD, Mitchell EAD, Buttler A (2015). Linking soil microbial communities to vascular plant abundance along a climate gradient. New Phytologist.

[ref-5] Breshears DD (2006). The grassland–forest continuum: trends in ecosystem properties for woody plant mosaics?. Frontiers in Ecology and the Environment.

[ref-6] Briggs JM, Knapp AK, Blair JM, Heisler JL, Hoch GA, Lett MS, McCarron JK (2005). An ecosystem in transition: causes and consequences of the conversion of mesic grassland to shrubland. BioScience.

[ref-7] Caporaso JG, Kuczynski J, Stombaugh J, Bittinger K, Bushman FD, Costello EK, Fierer N, Peña AG, Goodrich JK, Gordon JI, Huttley GA, Kelley ST, Knights D, Koenig JE, Ley RE, Lozupone CA, McDonald D, Muegge BD, Pirrung M, Reeder J, Sevinsky JR, Turnbaugh PJ, Waters WA, Widmann J, Yatsuneko T, Zaneveld J, Knight R (2010). QIIME allows analysis of high-throughput community sequencing data. Nature Methods.

[ref-8] Chen LY, Li H, Zhang PJ, Zhao X, Zhou LH, Liu TY, Hu HF, Bai YF, Shen HH, Fang JY (2015). Climate and native grassland vegetation as drivers of the community structures of shrub-encroached grasslands in Inner Mongolia, China. Landscape Ecology.

[ref-9] Chu HY, Gorgan P (2010). Soil microbial biomass, nutrient availability and nitrogen mineralization potential among vegetation-types in a low arctic tundra landscape. Plant and Soil.

[ref-10] Chu HY, Sun HB, Tripathi BM, Adams JM, Huang R, Zhang YJ, Shi Y (2016). Bacterial community dissimilarity between the surface and subsurface soils equals horizontal differences over several kilometers in the western Tibetan plateau. Environmental Microbiology.

[ref-11] Coetzee BWT, Tincani L, Wodu Z, Mwasi SM (2008). Overgrazing and bush encroachment by *Tarchonanthus camphoratus* in a semi-arid savanna. African Journal of Ecology.

[ref-12] Daims H, Lebedeva EV, Pjevac P, Han P, Herbold C, Albertsen M, Jehmlich N, Palatinszky M, Vierheilig J, Bulaev A, Kirkegaard RH, Von Bergen M, Rattei T, Bendinger B, Nielsen PH, Wagner M (2015). Complete nitrification by *Nitrospira* bacteria. Nature.

[ref-13] Edgar RC (2010). Search and clustering orders of magnitude faster than BLAST. Bioinformatics.

[ref-14] Eldridge DJ, Bowker MA, Maestre FT, Roger E, Reynolds JF, Whitford WG (2011). Impacts of shrub encroachment on ecosystem structure and functioning: towards a global synthesis. Ecology Letters.

[ref-15] Faith DP (1992). Conservation evaluation and phylogenetic diversity. Biological Conservation.

[ref-16] Gellie NJC, Mills JG, Breed MF, Lowe AJ (2017). Revegetation rewilds the soil bacterial microbiome of an old field. Molecular Ecology.

[ref-17] Gibbons SM, Lekberg Y, Mummey DL, Sangwan N, Ramsey PW, Gilbert JA (2017). Invasive plants rapidly reshape soil properties in a grassland ecosystem. mSystems.

[ref-18] Griffiths RI, Thomson BC, James P, Bell T, Bailey M, Whiteley AS (2011). The bacterial biogeography of British soils. Environmental Microbiology.

[ref-19] Gómez-Rey MX, Madeira M, Gonzalez-Prieto SJ, Coutinho J (2013). Soil C and N dynamics in a Mediterranean oak woodland with shrub encroachment. Plant and Soil.

[ref-20] Hardy OJ (2008). Testing the spatial phylogenetic structure of local communities: statistical performances of different null models and test statistics on a locally neutral community. Journal of Ecology.

[ref-21] Hart SC, DeLuca TH, Newman GS, MacKenzie MD, Boyle SI (2005). Post-fire vegetative dynamics as drivers of microbial community structure and function in forest soils. Forest Ecology and Management.

[ref-22] Hibbard KA, Archer S, Schimel DS, Valentine DW (2001). Biogeochemical changes accompanying woody plant encroachment in a subtropical savanna. Ecology.

[ref-23] Ho J, Chambers LG (2019). Altered soil microbial community composition and function in two shrub encroached marshes with different physicochemical gradients. Soil Biology and Biochemistry.

[ref-24] Jackson RB, Banner JL, Jobbágy EG, Pockman WT, Wall DH (2002). Ecosystem carbon loss with woody plant invasion of grasslands. Nature.

[ref-25] Knapp AK, Briggs JM, Collins SL, Archer SR, Bret-Harte MS, Ewers BE, Peters DP, Young DR, Shaver GR, Pendall E, Cleary MB (2008). Shrub encroachment in North American grasslands: shifts in growth form dominance rapidly alters control of ecosystem carbon inputs. Global Change Biology.

[ref-26] Kurc SA, Small EE (2004). Dynamics of evapotranspiration in semiarid grassland and shrubland ecosystems during the summer monsoon season, central New Mexico. Water Resources Research.

[ref-27] Langfelder P, Horvath S (2012). Fast R functions for robust correlations and hierarchical clustering. Journal of Statistical Software.

[ref-28] Langille MGI, Zaneveld J, Caporaso JG, McDonald D, Knights D, Reyes JA, Clemente JC, Burkepile DE, Vega Thurber RL, Knight R, Beiko RG, Huttenhower C (2013). Predictive functional profiling of microbial communities using 16S rRNA marker gene sequences. Nature Biotechnology.

[ref-29] Lett MS, Knapp AK (2003). Consequences of shrub expansion in mesic grassland: resource alterations and graminoid responses. Journal of Vegetation Science.

[ref-30] Liu JJ, Sui YY, Yu ZH, Shi Y, Chu HY, Jin J, Liu XB, Wang GH (2014). High throughput sequencing analysis of biogeographical distribution of bacterial communities in the black soils of northeast China. Soil Biology and Biochemistry.

[ref-31] Maestre FT, Bowker MA, Puche MD, Hinojosa MB, Martinez I, Garcia-Palacios P, Castillo AP, Soliveres S, Luzuriaga AL, Sanchez AM, Carreira JA, Gallardo A, Escudero A (2009). Shrub encroachment can reverse desertification in semi-arid Mediterranean grasslands. Ecology Letters.

[ref-32] McClaran MP, Moore-Kucera J, Martens DA, Van Haren J, Marsh SE (2008). Soil carbon and nitrogen in relation to shrub size and death in a semi-arid grassland. Geoderma.

[ref-33] McKinley DC, Blair JM (2008). Woody plant encroachment by *Juniperus virginiana* in a mesic native grassland promotes rapid carbon and nitrogen accrual. Ecosystems.

[ref-34] Meyer KM, Wiegand K, Ward D (2009). Patch dynamics integrate mechanisms for savanna tree–grass coexistence. Basic and Applied Ecology.

[ref-35] Paul EA, Clark FE (1996). Soil microbiology and biochemistry.

[ref-36] Peng H-Y, Li X-Y, Li G-Y, Zhang Z-H, Zhang S-Y, Li L, Zhao G-Q, Jiang Z-Y, Ma Y-J (2013). Shrub encroachment with increasing anthropogenic disturbance in the semiarid Inner Mongolian grasslands of China. Catena.

[ref-37] Poudel R, Jumpponen A, Schlatter D, Paulitz T, Gardener BM, Kinkel L, Garrett K (2016). Microbiome networks: a systems framework for identifying candidate microbial assemblages for disease management. Phytopathology.

[ref-38] Prescott CE, Grayston SJ (2013). Tree species influence on microbial communities in litter and soil: current knowledge and research needs. Forest Ecology and Management.

[ref-39] Purcell D, Sompong U, Yim LC, Barraclough TG, Peerapornpisal Y, Pointing SB (2007). The effects of temperature, pH and sulphide on the community structure of hyperthermophilic streamers in hot springs of northern Thailand. FEMS Microbiology Ecology.

[ref-40] Reynolds JF, Virginia RA, Kemp PR, de Soyza AG, Tremmel DC (1999). Impact of drought on desert shrubs: effects of seasonality and degree of resource island development. Ecological Monographs.

[ref-41] Rivest D, Rolo V, López-Díaz L, Moreno G (2011). Shrub encroachment in Mediterranean silvopastoral systems: Retama sphaerocarpa and Cistus ladanifer induce contrasting effects on pasture and Quercus ilex production. Agriculture, Ecosystems & Environment.

[ref-42] Schlesinger WH, Pilmanis AM (1998). Plant-soil interactions in deserts. Biogeochemistry.

[ref-43] Schlesinger WH, Reynolds JF, Cunningham GL, Huenneke LF, Jarrell WM, Ross VA, Whitford WG (1990). Biological feedbacks in global desertification. Science.

[ref-44] Scholes RJ, Archer SR (1997). Tree-grass interaction in savannas. Annual Review of Ecology and Systematics.

[ref-45] Segata N, Izard J, Walron L, Gevers D, Miropolsky L, Garrett WS, Huttenhower C (2011). Metagenomic biomarker discovery and explanation. Genome Biology.

[ref-46] Shi Y, Xiang XJ, Shen CC, Chu HY, Neufeld JD, Walker VK, Groganc P (2015). Vegetation-associated impacts on arctic tundra bacterial and microeukaryotic communities. Applied and Environmental Microbiology.

[ref-47] Smith DL, Johnson L (2003). Expansion of *Juniperus virginiana* L. in the Great Plains: changes in soil organic carbon dynamics. Global Biogeochemical Cycles.

[ref-48] Soliveres S, Eldridge DJ (2014). Do changes in grazing pressure and the degree of shrub encroachment alter the effects of individual shrubs on understorey plant communities and soil function?. Functional Ecology.

[ref-49] Staddon PL, Ramsey CB, Ostle N, Ineson P, Fitter AH (2003). Rapid turnover of hyphae of mycorrhizal fungi determined by AMS microanalysis of ^14^C. Science.

[ref-50] Stegen JC, Lin X, Konopka AE, Fredrickson JK (2012). Stochastic and deterministic assembly processes in subsurface microbial communities. ISME Journal.

[ref-51] Sul WJ, Asuming-Brempong S, Wang Q, Tourlousse DM, Penton CR, Deng Y, Rodrigues JLM, Adiku SGK, Jones JW, Zhou JZ, Cole JR, Tiedje JM (2013). Tropical agricultural land management influences on soil microbial communities through its effect on soil organic carbon. Soil Biology and Biochemistry.

[ref-52] Throop HL, Archer SR (2008). Shrub (*Prosopis velutina*) encroachment in a semidesert grassland: spatial-temporal changes in soil organic carbon and nitrogen pools. Global Change Biology.

[ref-53] Trumbore SE (1997). Potential responses of soil organic carbon to global environmental change. Proceedings of the National Academy of Sciences of the United States of America.

[ref-54] Van Vegten JA (1984). Thornbush invasion in a savanna ecosystem in eastern Botswana. Vegetatio.

[ref-55] Wallenstein MD, McMahon S, Schimel J (2007). Bacterial and fungal community structure in Arctic tundra tussock and shrub soils. FEMS Microbiology Ecology.

[ref-56] Webb CO (2000). Exploring the phylogenetic structure of ecological communities: an example for rain forest trees. American Naturalist.

[ref-57] Xiang XJ, Gibbons SM, Li H, Shen HH, Fang JY, Chu HY (2018). Shrub encroachment is associated with changes in soil bacterial community composition in a temperate grassland ecosystem. Plant and Soil.

[ref-58] Yannarell AC, Menning SE, Beck AM (2014). Influence of shrub encroachment on the soil microbial community composition of remnant hill prairies. Microbial Ecology.

[ref-59] Zhang Z, Wang S-P, Nyren P, Jiang G-M (2006). Morphological and reproductive response of *Caragana microphylla* to different stocking rates. Journal of Arid Environments.

[ref-60] Zhang XF, Xu SJ, Li CM, Zhao L, Feng HY, Yue GY, Ren ZW, Cheng G (2014). The soil carbon/nitrogen ratio and moisture affect microbial community structures in alkaline permafrost-affected soils with different vegetation types on the Tibetan Plateau. Research in Microbiology.

[ref-61] Zhou JZ, Xue K, Xie JP, Deng Y, Wu LY, Cheng XL, Fei SF, Deng SP, He ZL, Van Nostrand JD, Luo YQ (2012). Microbial mediation of carbon-cycle feedbacks to climate warming. Nature Climate Change.

[ref-62] Zuur AF, Leno EN, Elphick CS (2010). A protocol for data exploration to avoid common statistical problems. Methods in Ecology and Evolution.

