# Supplementary material for: Proximate grassland and shrub-encroached sites show dramatic restructuring of soil bacterial communities
Source: PeerJ. 2019 Jul 19;7:e7304. doi: 10.7717/peerj.7304 (PMC6644630; doi:10.7717/peerj.7304)
Supplement: Supplemental Information 2 [file peerj-07-7304-s002.docx]

**Supporting Information**

**Soil analyses**

Soil pH was measured in a soil water suspension (1:5 wt/vol), and soil moisture (SM) was measured gravimetrically. Standard methods were used for measuring soil total P (TP, *Bowman, 1988*), total C (TC) and total N (TN) (*Walkley and Black, 1934*). Soil inorganic carbon (SIC) was measured by the neutralization titration method. Soil organic carbon (SOC) = TC - SIC. Soil dissolved organic C (DOC), total dissolved N (TDN), and mineral N were measured after extraction by adding 50 ml of 0.5 M K_2_SO_4_ to 10 g fresh soil. Ammonium (NH_4_^+^) and nitrate (NO_3_^-^) contents in the extracts were determined colorimetrically by automated segmented flow analysis (Bran + Luebbe AAIII, Germany). A TOC-TN analyzer (Shimadzu, Kyoto, Japan) was used to measure DOC and TDN. Dissolved organic N (DON) was calculated as follows: DON = TDN - NH_4_^+^ - NO_3_^-^.

**Soil DNA extraction and purification**

DNA extractions were carried out on 0.5 g fresh soil according to the manufacturer’s instructions (FastDNA® SPIN Kit for soil, MP Biomedicals, Santa Ana, CA). DNA was purified using a PowerClean® DNA Clean-Up Kit (MO BIO Laboratories, Carlsbad, CA, USA) to remove PCR inhibitors. The extracted DNA was dissolved in 50 μl of elution buffer, quantified by NanoDrop ND-1000 (Thermo Scientific, USA), and stored at -20 °C.

**PCR and amplicon library preparation**

An aliquot (50 ng) of purified DNA from each sample was used as template for amplification. Primer sets F515/R907 equipped with sequencing adapters and unique identifier tags were used to amplify the V4-V5 hypervariable regions of the bacterial 16S rRNA genes fragments (*Biddle et al., 2008*) for the Illumina MiSeq platform (PE 300) at Majorbio (Shanghai, China). PCR was carried out in 50 μl reaction mixtures containing each deoxynucleoside triphosphate at a concentration of 1.25 mM, 1 μl of forward and reverse primers (20 mM), 2 U of Taq DNA polymerase (TaKaRa, Japan), 2 mM of MgCl_2_, and 50 ng of DNA. The following cycling parameters were used: 35 cycles of denaturation at 94 °C for 45 s, annealing at 55 °C for 45 s, and extension at 72 °C for 45 s; with a final extension at 72 °C for 10 min. To check for contamination, PCR negative controls were performed without added DNA template. Negative PCR controls did not contain detectable PCR product, and were not processed for sequencing. Triplicate reaction mixtures per sample were pooled together and purified using an agarose gel DNA purification kit (TaKaRa). The PCR products were pooled in equimolar amounts (10 pg for each sample) before sequencing.

**References:**

**Biddle JF, Fitz-Gibbon S, Schuster SC, Brenchley JE, House CH.** 2008. Metagenomic signatures of the Peru Margin subsea floor biosphere show a genetically distinct environment. *Proceedings of the national academy of sciences of the United States of America* **105**: 10583-10588.

**Bowman RA.** 1988. A rapid method to determine total phosphorus in soils. *Soil Science Society of America Journal* **52**: 1301-1304.

**Walkley A, Black IA.** 1934. An examination of the Degtjareff method for determining soil organic matter, and a proposed modification of the chromic acid titration method. *Soil Science* **37**: 29-38.

Table S1: Summary of the main soil properties in grassland and shrub-encroached soils. The values in brackets represent the standard deviation of the mean. Letters following brackets represent significant differences from pairwise t-test (*P* < 0.05).

| Variables | Grassland soils | Shrub-encroached soils |
| --- | --- | --- |
| Soil pH | 8.40 (0.72)^a^ | 8.42 (0.68)^a^ |
| Soil moisture (%) | 7.87 (1.27)^a^ | 7.99 (1.00)^a^ |
| NH_4_^+^-N (mg/kg) | 1.67 (0.33)^a^ | 2.21 (1.03)^a^ |
| NO_3_^-^-N (mg/kg) | 1.36 (0.60)^b^ | 3.43 (1.71)^a^ |
| Dissolved organic C (mg/kg) | 40.4 (8.2)^b^ | 65.5 (17.4)^a^ |
| Dissolved organic N (mg/kg) | 3.04 (0.68)^a^ | 4.58 (2.85)^a^ |
| Total carbon (mg/g) | 0.76 (0.09)^b^ | 1.04 (0.22)^a^ |
| Total nitrogen (mg/g) | 0.10 (0.01)^b^ | 0.12 (0.02)^a^ |
| Total phosphorus (mg/g) | 0.26 (0.02)^b^ | 0.29 (0.03)^a^ |
| Soil inorganic carbon (mg/g) | 0.02 (0.02)^a^ | 0.03(0.02)^a^ |
| Soil organic carbon (mg/g) | 0.74 (0.08)^b^ | 1.01 (0.22)^a^ |


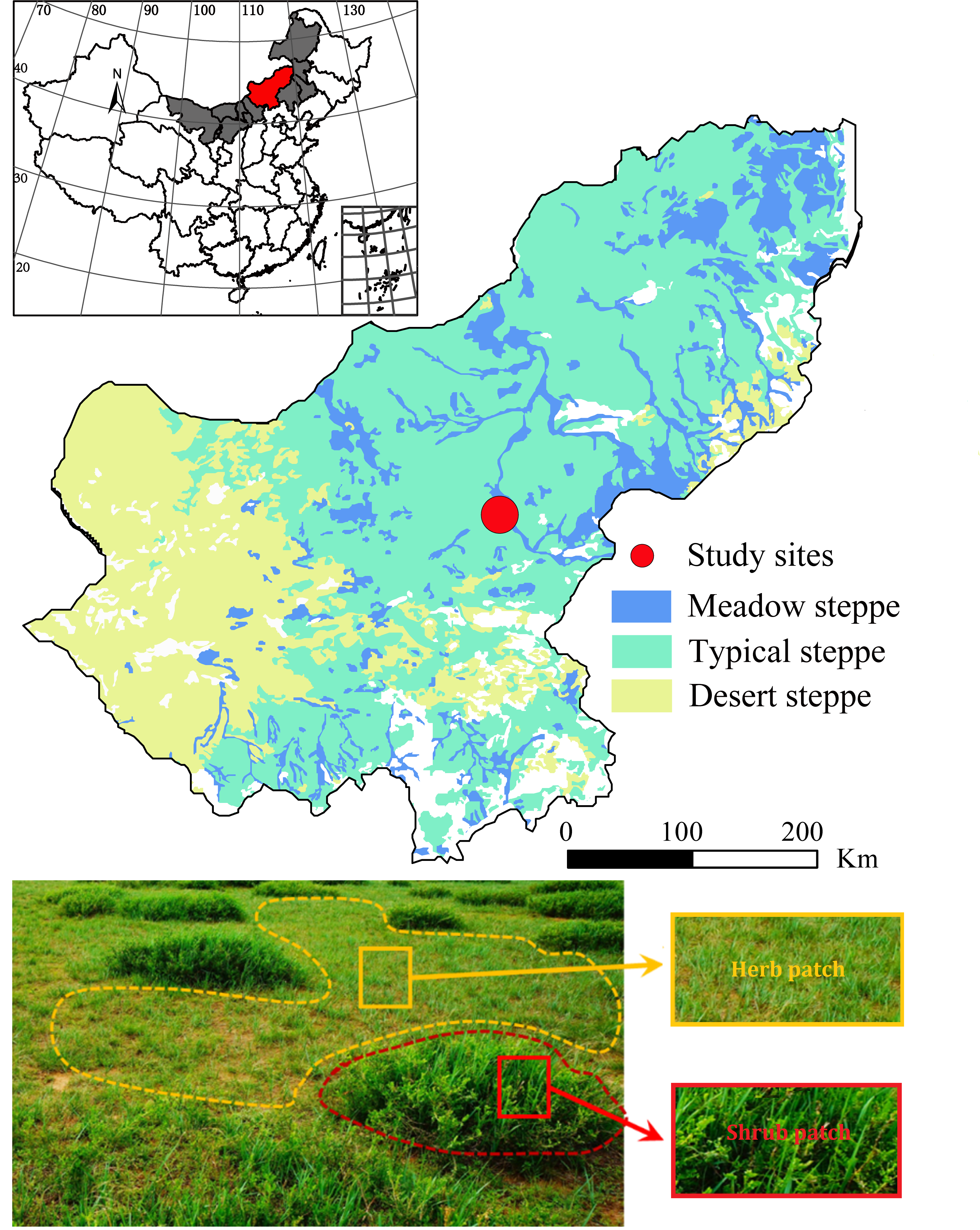
Figure S1: The schematic figure of sampling locations.


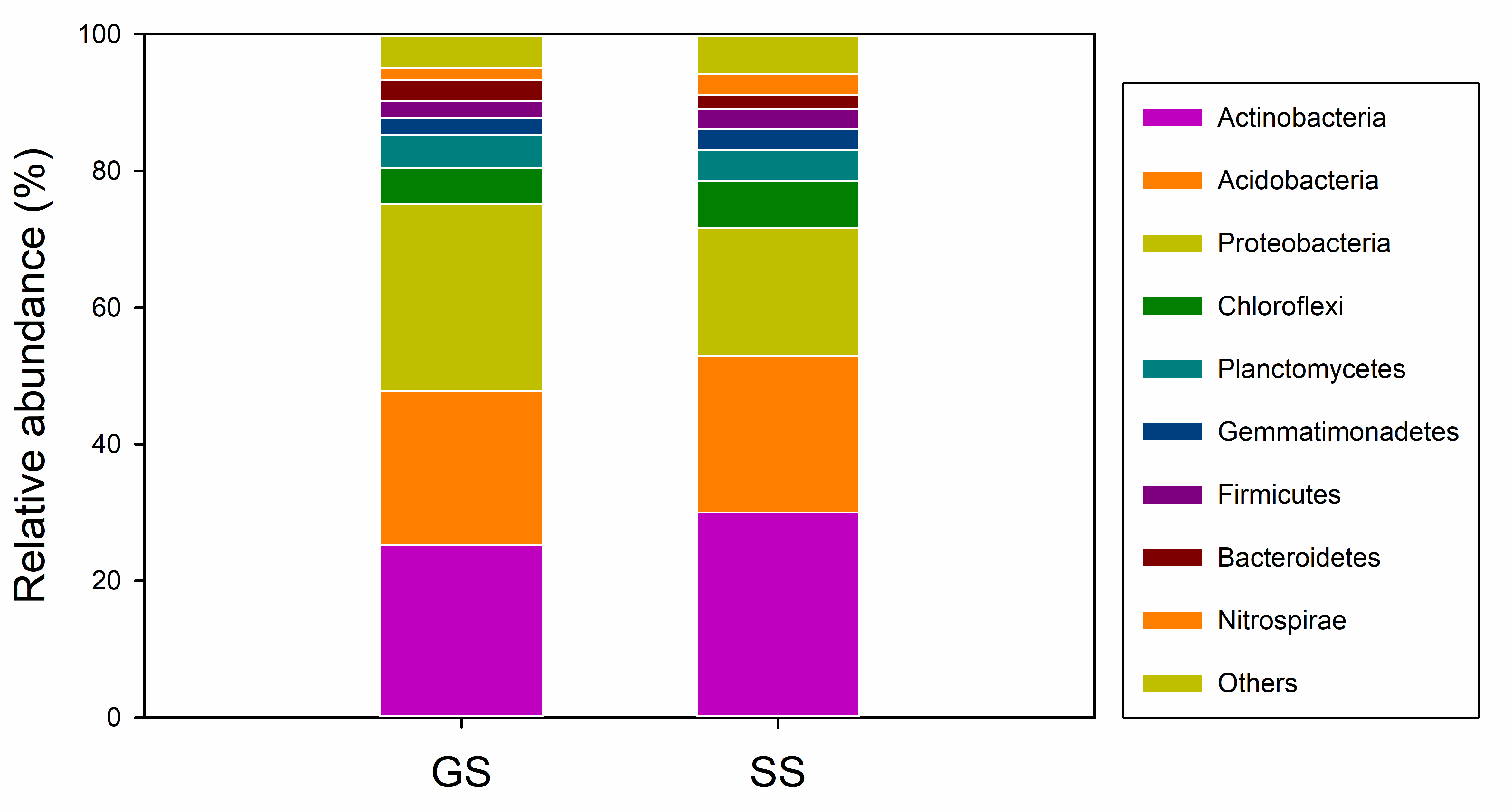
Figure S2: Relative abundances of the dominant bacterial phyla in soils. Relative abundances are based on the proportional frequencies of DNA sequences that could be classified at the phylum level. GS: control grassland soil; SS: shrub-encroached soil.


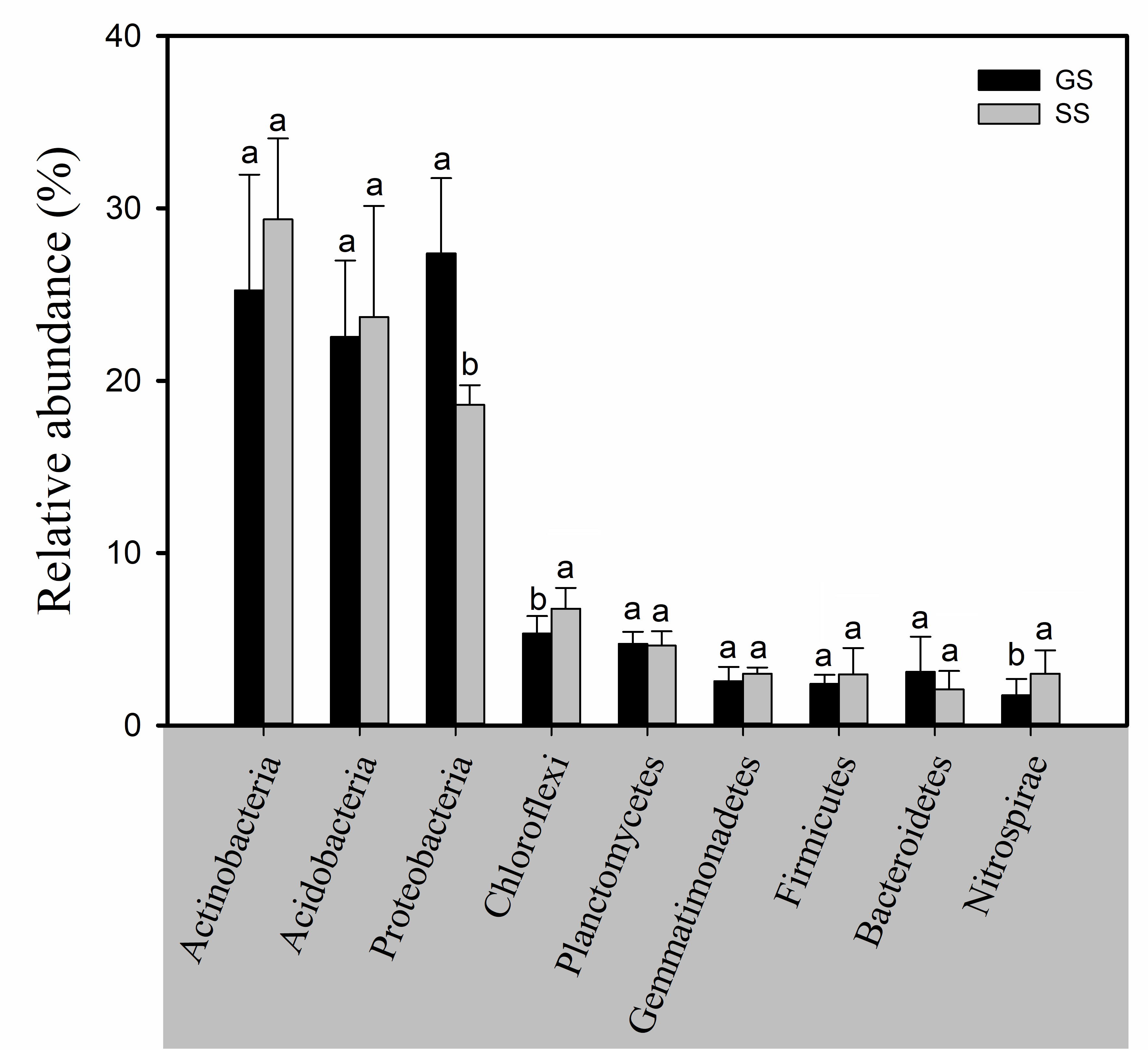
Figure S3: Relative abundances of the dominant bacterial phyla in grassland and shrub-encroached soils. Bars represent mean; error bars denote standard deviation; letters above bars represent significant differences from pairwise t-test (*P* < 0.05). GS: control grassland soil; SS: shrub-encroached soil.


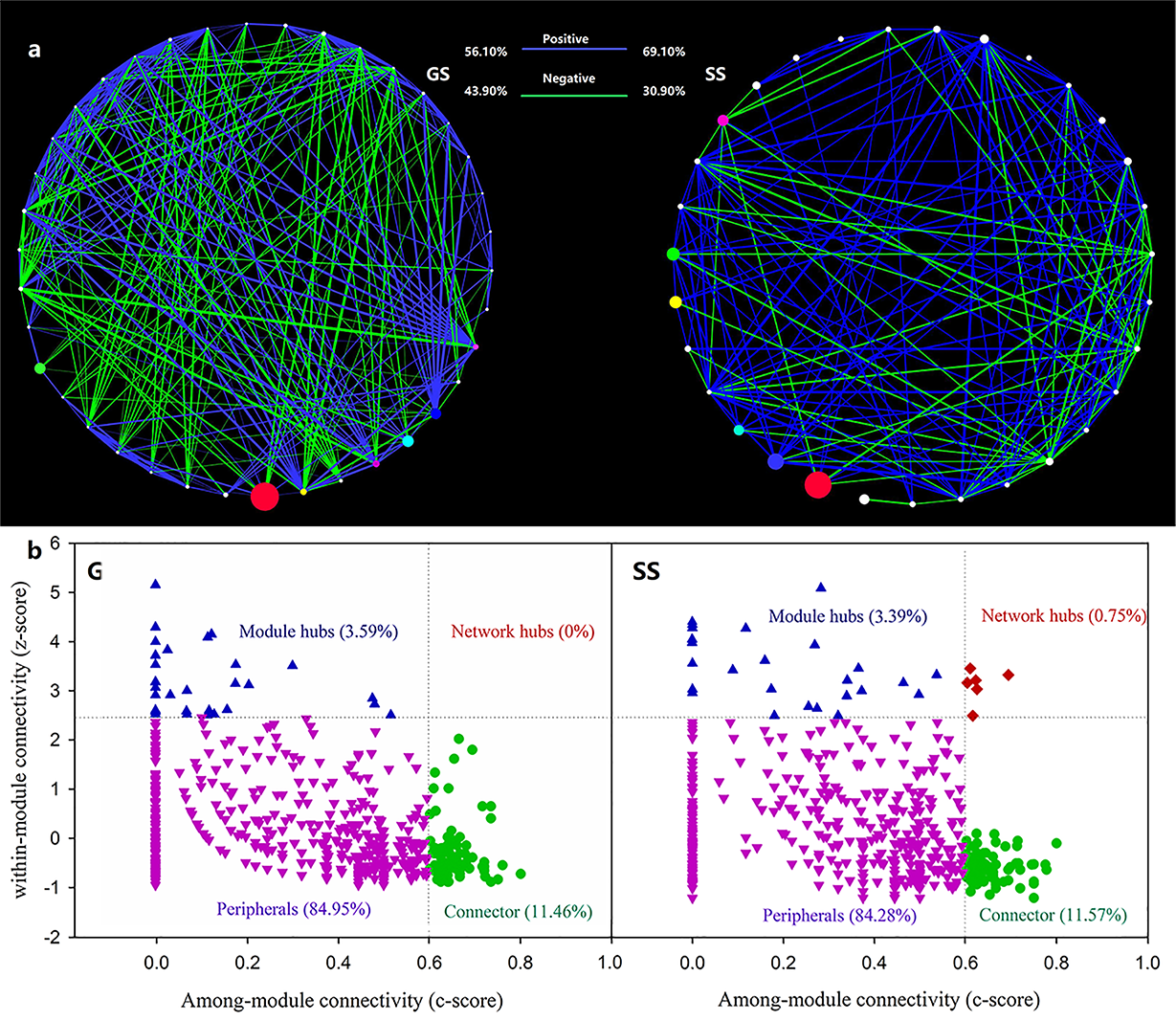
Figure S4: The co-occurrence network structure of bacterial community (a); and network roles of module feature (b). GS: control grassland soil; SS: shrub-encroached soil. Network hubs that are highly connected both in general and within a module ( z-score > 2.5; c-score > 0.6), module hubs that are highly connected only within a module (z-score > 2.5; c-score < 0.6), connectors that are highly connected only link modules (z-score < 2.5; c-score > 0.6) and peripherals that have few links to other species (z-score < 2.5; c-score < 0.6).


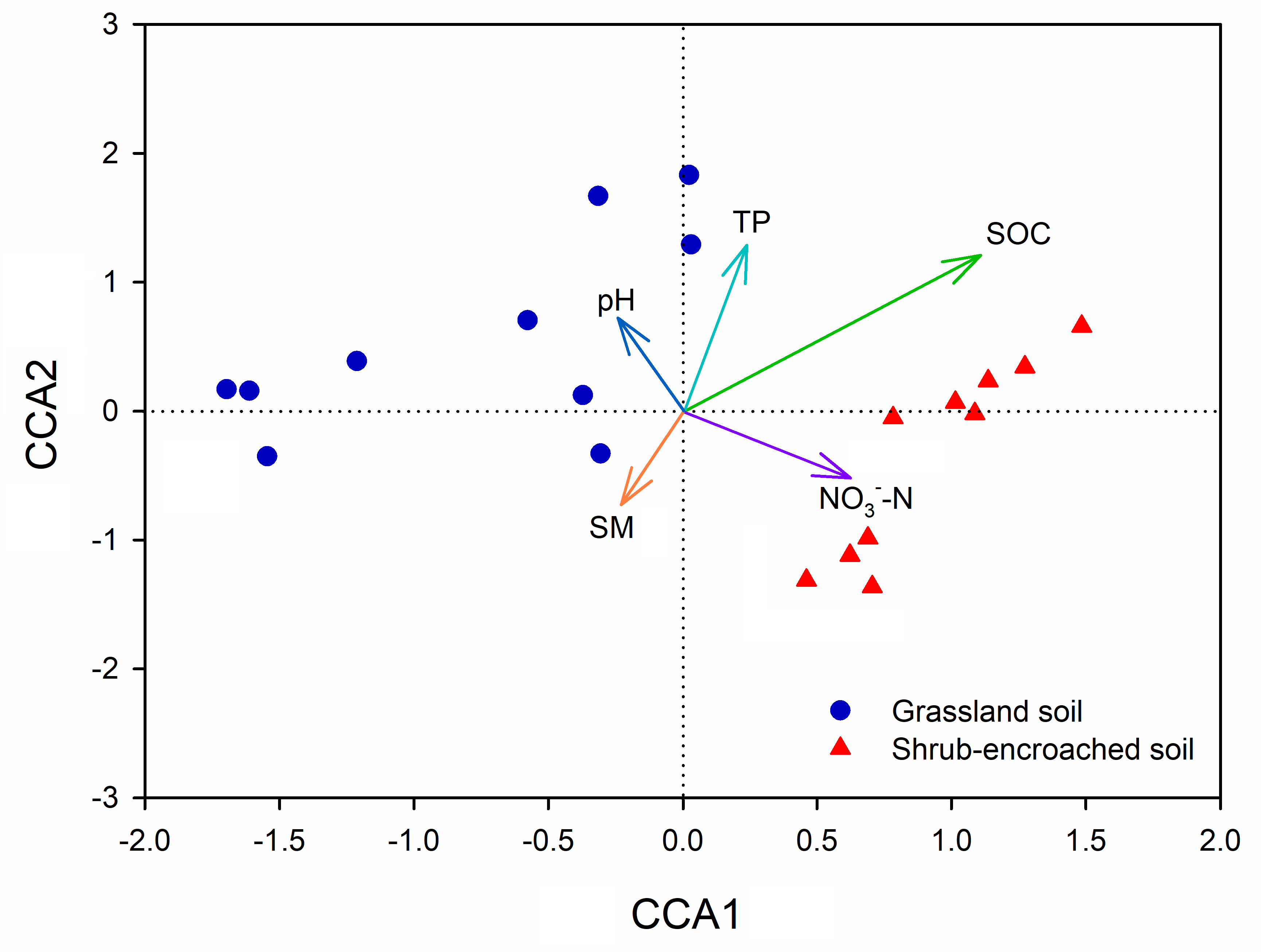
Figure S5: Canonical correspondence analysis (CCA) depicting the effect of soil properties on bacterial community composition. SOC: soil organic carbon; TP: total phosphorus; SM: soil moisture.


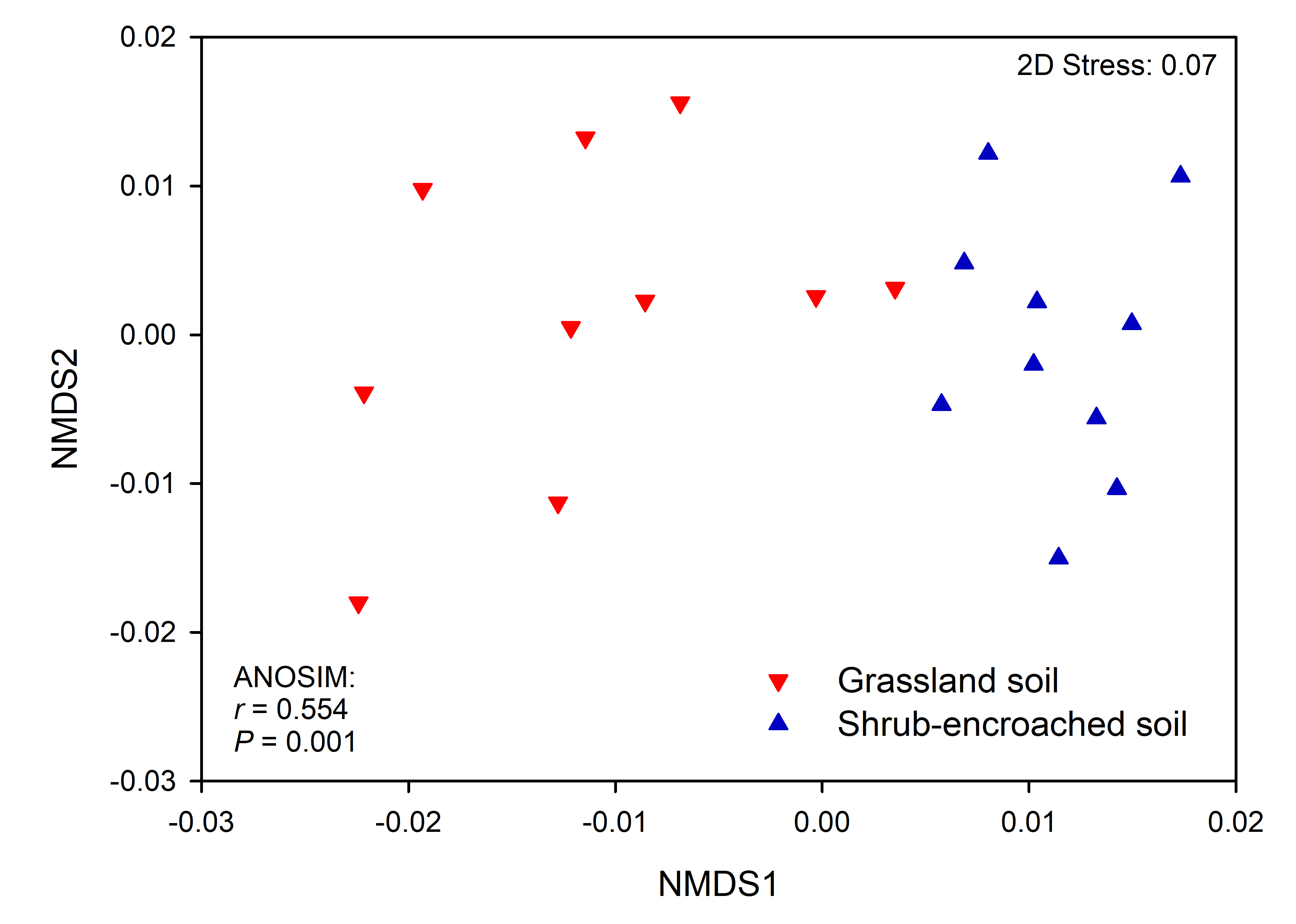
Figure S6: Non-metric multidimensional scaling (NMDS) plot showing the predicted metabolic function of bacterial community in grassland and shrub-encroached soils of Inner Mongolia.
